# The US Caselaw as a living system

**DOI:** 10.1371/journal.pone.0324386

**Published:** 2025-05-23

**Authors:** Carlos G.O. Fernandes, Erneson A. Oliveira, Rilder S. Pires, João A. Monteiro Neto, J. Ernesto Pimentel Fh., José S. Andrade Jr., Vasco Furtado

**Affiliations:** 1 Programa de Pós Graduaç ao em Informática Aplicada, Universidade de Fortaleza, Fortaleza, Ceará, Brazil; 2 BNB - Banco do Nordeste do Brasil S.A., Fortaleza, Ceará, Brazil; 3 Laboratório de Ciência de Dados e Inteligência Artificial, Universidade de Fortaleza, Fortaleza, Ceará, Brazil; 4 Mestrado Profissional em Ciências da Cidade, Universidade de Fortaleza, Fortaleza, Ceará, Brazil; 5 Centro de Ciências Jurídicas, Universidade de Fortaleza, Fortaleza, Ceará, Brazil; 6 Programa de Pós Graduaç ao em Ciências Jurídicas, Universidade Federal da Paraíba, Jo ao Pessoa, Paraíba, Brazil; 7 Departamento de Física, Universidade Federal do Ceará, Fortaleza, Ceará, Brazil; 8 ETICE - Empresa de Tecnologia da Informaç ao do Ceará, Fortaleza, Ceará, Brazil; Indian Statistical Institute, Interdisciplinary Statistical Research Unit, INDIA

## Abstract

This study presents an innovative exploration of the American Caselaw database, encompassing more than five million legal cases spanning three centuries of American history. Using complex network analysis, we reveal the organic nature of the US Caselaw, fundamentally anchored in common law. Through analysis of citation and bibliographic coupling networks, we shed light on the system’s internal structure, unveiling communities delineated by regional, federal jurisdiction, and clustering based on similar legal citations. Our research uncovers a remarkable allometric relationship between the activity of judges and the legal case citations, reflecting the analogy between metabolic rate and body mass correlation observed in biological organisms. Furthermore, our results show a consistent self-similar characteristics of the communities and their maximum spanning trees, which also provides relevant insight into the origin of the allometric behavior. This analysis not only reveals the US Caselaw as a “living” entity but also sets a precedent in Caselaw-based judicial system studies, reinforcing the notion of its dynamic, organic functionality in the realm of analyzing complex legal systems.

## Introduction

What is life? According to Koshland [1], life is an organized unit based on seven pillars: Program, Improvisation, Compartmentalization, Energy, Regeneration, Adaptability, and Seclusion (or PICERAS, for short). Koshland’s definition of life is only one among many of the current scientific answers for such primordial philosophical questioning [2, 3]. This quest to understand life often intersects with the exploration of fundamental concepts, particularly those related to energy and its manifestations. Indeed, the concept of energy, more specifically, the concept of metabolism, seems to permeate the majority of life definitions [4]. This pervasive focus on energy and metabolism in defining life pushes us towards universal principles that sustain living systems, suggesting that Kleiber’s law [5] could be used as an important criterion to define systems that behave as living organisms. In his seminal study, Kleiber showed that the basal metabolic rate (*Y*) and the body mass (*X*) of a large range of animals are related by an allometric scaling given by the following power law:

$$Y=aX^{\beta },$$ (1)

where β is the allometric exponent below 1 and *a* is a prefactor (constant). Often, the value of 3/4 is adopted for this exponent due to the ubiquity of the quarter-power scale in biology, however, the value of this exponent, as well as its origin, is the subject of theoretical debates that suggest that the value 2/3 could also be adopted [5–8]. An explanation for the origin of the power law shown in Eq 1 is the existence of space-filling fractal networks of branching tubes that allow the transport of essential materials through the living organism, as evidenced by the characteristics of vertebrate cardiovascular and respiratory systems, plant vascular systems, insect tracheal tubes, and other distribution networks [9].

Bettencourt *et al*. [10–12] demonstrated that, similar to biological systems, US cities exhibit allometric scaling with population size. Accordingly, human activities generally fall into three distinct scaling categories based on the allometric exponent value: isometric or linear behavior (β=1), sublinear allometric behavior (β<1), and superlinear scaling (β>1). The first aligns with the proportional scaling of individual needs such as jobs and housing in relation to population, while the sublinear scaling indicates economies of scale, as seen in fewer gasoline stations, shorter electrical cables, and less road surface per capita. Conversely, superlinearity emerges when social activities significantly impact urban metrics. This is evident not only in larger cities enhancing productivity and quality of life more than proportionally, as observed in higher incomes, GDP, and innovation rates, but also in the increase of negative indicators such as crime [12–14] and carbon dioxide emissions [15].

Regarding the American Judicial System, the United States adopts the common law, a legal framework that relies on court decisions and precedents. It means that legal decisions from previous cases help guide current judgments, and judges play a significant role in interpreting and applying the law. Unlike civil law, which is chiefly based on statutes and codes rather than a scattered historic collection of texts and proceeding values, the common law system develops through the practical application of law according to the specific circumstances of each legal case [16, 17]. The common law is an evolving system in which new judicial decisions can change or expand the interpretation and application of the law. Such adaptive behavior is compared to Darwin’s ideas on the biological history of living organisms [18, 19]. Edward P. Thompson’s approach [20] aptly reinstated Karl Marx’s interpretation of Charles Darwin’s Theory of Evolution, demonstrating that the significant contribution of Darwinian theory lies in the perception of transformation and changes of living beings, thus emphasizing its predominantly historical characteristic.

Elkins *et al*. [21] examined constitutions from countries worldwide and concluded that the process of constitutional crisis and death is akin to a biological process, the “equivalent of a description in a medical textbook of the breakdown of vital organs, complete with detailed micro information about tissue and cells”. They also added: “At a more remote level, however, our theory needs to connect these processes to genetic predispositions, activities, or conditions that might increase the risk of breakdown”.

In recent years, an increasing number of studies have been proposed to characterize legal systems through mathematical models [22–29], yet none have revealed results suggesting that legal systems exhibit properties like those of living organisms. Here, we propose that what we perceive as a “living judicial system” is, in fact, a dynamic set of “organisms.” These organisms are represented by communities of legally similar cases, identified through citation correlations that capture thematic, jurisdictional, and legal features of the cases. Like biological organisms, these communities exhibit dynamic behaviors and structural properties that reflect their adaptive and self-organizing nature.

By analyzing the citation patterns between cases, we uncover two key properties of the judicial system: (1) scaling laws, which govern the relationships between components of the system, and (2) self-similarity, observed within the communities themselves. By examining scaling relationships, we identify allometric scaling laws that, analogous to living systems, describe the interplay between the system’s “metabolism” (judicial activity) and the “mass” of its components (the volume of citations within communities). Similarly, the fractal nature of the citation networks, as revealed through the Maximum Spanning Trees (MSTs) of these communities, emphasizes the self-organizing properties of the system.

## Network models

**Fig 1:**
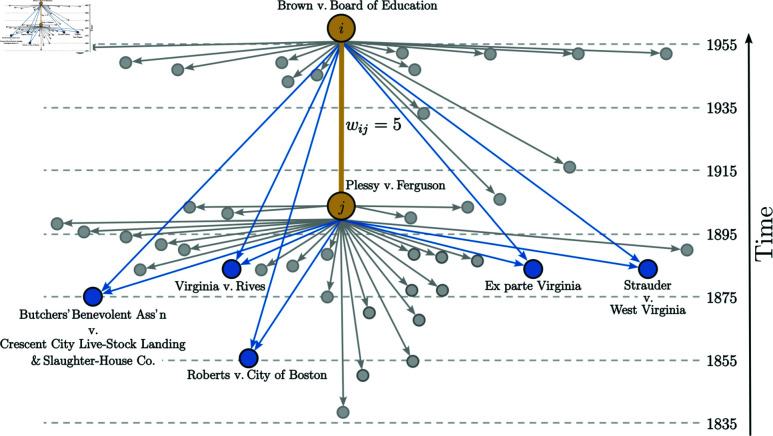
Outline of the citation and bibliographic coupling networks. Arrows represent citations made by two legal cases. The arrows in blue represent the citations (directed edges) in common between the cases, and the ones in gray represent the remaining citations of the legal cases. The thick orange line represents the undirected edge of the bibliographic coupling between these two cases, which has a weight *w*_*ij*_ = 5 (since the two cases have 5 citations in common). The two vertices in orange represent two very emblematic decisions in American courts. The vertex *j*, *The Plessy v. Ferguson*, 163 U.S. 537 (1896), is the decision that created the “separate but equal” doctrine that allowed racial segregation. The other landmark decision is vertex *i*, *Brown v. Board of Education of Topeka*, 347 U.S. 483 (1954), which defined the reversal of the “separate but equal” doctrine.

We propose a methodology based on two classical network models [30]: (i) citation network and (ii) bibliographic coupling network, emphasizing the second model. We define a citation network over the Caselaw Access Project (CAP) dataset (see Methods), where the vertices are legal cases and the directed edges are citations (from newer legal cases to older legal cases), as shown in Fig 1. To ensure an acyclic network, we eliminate duplicate edges that occurs for exceptional circumstance. Furthermore, we construct a weighted bibliographic coupling network within the *Largest Weakly Connected Component (LWCC)* of the *citation network* to save memory and processing time as much as possible. We emphasize that this approach does not change our results. Although the citation network is the natural framework for studying growth processes, here, we are interested in measuring the similarity between legal cases. For this reason, it is appropriate to define the bibliographic coupling network. For the *bibliographic coupling network*, the vertices *i* are also legal cases, and the undirected weighted edges (*i*,*j*,*w*_*ij*_) are representations that model the similarity between two legal cases, *j* and *i*, where each weight *w*_*ij*_ is the number of legal cases that both cite, as also shown in Fig 1. Formally, we can obtain the *bibliographic coupling network* through its adjacency matrix,

$$B=A^{T}A,$$ (2)

where *A* is the adjacency matrix from the LWCC of the *citation network*. It is important to point that such matrix multiplication demand a huge computational effort for large matrices, even for sparse ones. Finally, we consider the *Largest Connected Component (LCC)* of the *bibliographic coupling network* as our object of study to analyse communities of similar legal cases.

It is important to highlight that the bibliographic coupling network has many more edges than the citation network, as will be shown in the next section. For this reason it ends up being opportune to eventually work with subgraphs of the network, precisely we will study the communities of the network and eventually the *maximum spanning tree* of these structures. The *Maximum Spanning Tree* (MST) can be defined similarly to how we define the *minimum spanning tree* [31], as the acyclic subgraph that includes all vertices and whose sum of edge weights is maximum. Thus, by definition, the MST resembles the space-filling condition required by West *et al*. [9]. The fractal-like characteristic of this structure will be investigated in more detail in the next section.

## Methods

The workflow is composed of sequential steps as follows: (i) Construction of the Citation Network, which provides the foundational structure for understanding the interactions and dependencies between cases; (2) Generation of the Bibliographic Coupling Network, which highlights the degree of similarity among cases based on their references; (3) Community detection in the Bibliographic Coupling Network, forming the "organisms" of the US Caselaw in our analogy; (4) Analysis of Scaling Laws in the network, providing insight into the systemic properties of the judicial network; (5) Construction/Selection of a Maximum Spanning Tree (MST) enabling a more computationally efficient analysis of structural patterns within the network; (6) Calculation of the Fractal Dimension of the MSTs, which allows us to assess whether the network exhibits structural patterns similar to fractal systems; (7) Extraction of judges from the communities for providing additional context regarding the human element that impacts the network structure and behavior; (7) Uncovering allometric relationships similar to Kleiber’s Law in biological systems. Following this step-by-step approach, we incrementally constructed and analyzed the networks to uncover properties of the US Caselaw that align with our hypothesis that it behaves like living organisms. The details of the datasets and the tools used in these steps are described below.

### Datasets

#### Courtlistener.

CourtListener is a website and dataset, part of the FreeLaw project (https://free.law/about) that provides access to millions of legal decisions from American courts. This data is fed by public sources and contains various information about the processes and the judges who participated in them. The data was accessed via API (https://www.courtlistener.com/help/api/rest/) to retrieve the judges’ names. In total, 16,163 names of judges were extracted with information on race, sex, party affiliation and positions held by the person.

#### Caselaw access project.

The Caselaw Access Project (CAP) seeks to provide public and online access to all published US court decisions [32]. The CAP is maintained by the Harvard Law School with legal cases that range from 1658 to 2020. For each legal case, it is provided a set of retrieved information, such as name, decision date, jurisdiction, court, opinion, and citations. The basic figures for the CAP dataset are shown in Table 1. There are 7 tables: Cases, Jurisdiction, Court, Volume, Reporter, Judges and Citation. “Cases” and “Citation” are the largest with 6,920,598 and 4,470,376 rows respectively.

**Table 1:** Basic data metrics.

Table	Fields	Rows	Format	Example of Fields
Cases	26	6,920,598	JSON / Html	id, url, name, decision_date, court, jurisdiction, casebody
Jurisdiction	6	63	JSON / Html	id, name_long, name
Court	7	3,501	JSON / Html	name_abbreviation, id, name
Volume	16	40,704	JSON / Html	volume_number, barcode
Reporter	8	651	JSON / Html	full_name, id
Judges^*^	3	13,678	CSV	name, case
Citation^**^	2	4,470,376	CSV	vertice, adjacency_list

^*^ We used a semi-automatic method to identify judges (see Methods)

^**^ This information is provided as an *adjacency list* that describes the set of neighbors for each vertex

Basic information about the largest tables. Note that “Cases” and “Citation” have millions of rows.

### The process of judges name extraction

In Caselaw, cases are structured in Sections and Subsections. Judges’ names is in the casebody’s Author subsection or linked to the Opinion and Judge subsections (see S1 Fig for details of that structure). The former lists the rapporteur, while the latter includes other judges. Of the 6,930,777 decisions in the database, 94% feature at least one subsection. However, these often lack actual names. The omission of judges’ names in opinions is typically due to the opinion’s nature or court preferences rather than district-specific rules, leading to unnamed judges in some instances. There are circumstances in which an opinion may not contain the names of individual judges, as following:

- *Per Curiam* Decisions: A *per curiam* decision is a unanimous ruling issued by an entire appellate court. This term or phrases like “all the judges concur”, “all concur”, etc. indicate a collective decision, often without attributing the judgment to specific judges. S2 Fig contains details about the most frequent terms associated with such anonymous decisions.
- Unanimous Decisions: When a decision is unanimous and lacks dissenting or concurring opinions, the court may opt not to attribute the decision to any particular judge, reflecting the collective agreement of the bench;
- Procedural Orders: These are often brief, administrative, or procedural directives that do not require extensive legal analysis. As such, they’re typically issued without detailing individual authorship;
- Summary Rulings: For cases that deal with routine matters or well-established laws, courts might issue summary rulings. These are concise decisions that often omit the names of the judges to reflect the straightforward nature of the ruling.

After treating the processes where the cases had a unanimous collegiate decision, 78% of decisions remained for the exact names of the judges to be extracted. To do this, we customized Spacy’s Named Entity Recognition (NER) model (https://spacy.io/models/en#en_core_web_lg). The customization was done based on a sample of Caselaw where the judges’ names were previously labeled. Labeling was done via a combination of automated and manual techniques. Here’s a step-by-step breakdown of the methodology:

1. Grouping Similar Names: The process began by identifying and grouping the same names that appear in the Author and Judges subsections of the documents. For instance, “DAVIDSON, Presiding Judge” appeared in 2,123 cases, and “LATTIMORE, Judge” appeared in 5,120 cases.
2. Creating Unique Text Patterns: Through this initial grouping, 1,162,475 distinct text patterns were identified. These text patterns represent different ways judges’ names and titles might appear in the documents.
3. Normalization: To further refine the data, all text patterns were made lowercase, and invalid characters were removed. This step reduced the number of distinct text patterns to 974,948.
4. Grouping by Text Size and Applying Regular Expressions (Regex):
5. Half of them (483,345) are between 3 and 50 characters in length. For each of these sizes, a specific form of regular expression (regex) to extract the judges names was programmed. Some groups required more than one regex formula so that all texts had judges extracted. S1 File describes all regex used.
6. For texts longer than 50 characters, totaling 699 other groups, the variability did not allow building regex for the entire universe. A manual annotation strategy was adopted in a random sample of 1% of the texts. At least one example from each group was selected, thus totaling 5,469 texts for manual annotation by an expert.
7. Manual Annotation: An expert manually annotated these selected texts, identifying and recording the judges’ names.

This methodology demonstrates a hybrid approach to text mining, where automated techniques are used to handle the bulk of the data, and manual intervention is applied where the automated methods fall short. The outcome of this process would be a more accurate and comprehensive database of judges’ names as they appear across a vast body of legal documents.

The complete dataset of 491,814 texts was utilized to refine the Spacy *en_core_web_lg* NER model, specifically aimed at identifying and extracting judge names. In this process, 80% of the cases were designated for the training phase, with the remaining 20% reserved for testing the model’s efficacy. After undergoing 100 iterations with a dropout rate set at 0.3, the model reached f-measure of 0.996, boasting a precision of 0.994 and a recall of 0.997 specifically for the “PERSON” entity category, which in this context, pertains to judges. The trained NER model successfully extracted 116,786 distinct judge names from the database, with only 0.06% of cases not yielding any judge names.

Despite these successes, a significant number of names were plagued by spelling errors, primarily due to *Optical Character Recognition* (OCR) inaccuracies. To rectify this, a new stage of data cleaning was implemented. The strategy employed involved comparing the erroneous names against a repository of correctly spelled names from the Free Law Database. The assumption here was that the misspellings, while frequent, would not consistently misrepresent the same name in the same way. For example, a judge named “Smith” might occasionally be recorded as “Sm1th.” To address this, we used the Jaro-Winkler algorithm, which calculates the similarity between two strings, providing a normalized score between 0 and 1—the closer to 1, the greater the similarity. Using this method, we could match and correct many of the misspelled names.

In 15% (or 2,982) of the unique judge names extracted were not present in the Free Law Database, yet they might still be correctly spelled. To discern which of these names were likely accurate, we employed a heuristic based on occurrence frequency—names appearing more than 15 times were presumed to be correct, based on the logic that consistent misspellings are improbable. This approach not only validated frequently occurring names but also provided a reference for correcting less common ones. This rigorous cleaning process corrected 1,571 names.

In the final phase of this process, we integrated the cleaned and validated names with additional data from the Free Law database. This integration aimed to provide contextual details such as birth, death, or retirement dates, crucial for disambiguating judges with common names and determining their active period during the relevant cases. Through this reconciliation process, we managed to match 48% of the 17,576 distinct names, enhancing the database’s accuracy and utility.

### Community detection algorithm

We use a parallelized version of the Louvain algorithm, called Parallel Louvain Method (PLM) [33], to perform the detection of communities in the bibliographic coupling network. PLM operates in two distinct stages: initially, it seeks to optimize the modularity of the network by identifying sets of strongly interconnected nodes, forming local communities, that is, groups that are densely linked together compared to the rest of the network. Subsequently, it reorganizes the network structure, transforming the communities detected in the previous step into individual nodes. For all parameters, we used the default values provided by networkit.community.PLM module in the Networkit toolkit (https://networkit.github.io/) [34, 35]. We explored the variation of the multi-resolution modularity parameter to examine and analyze the number and size of identified communities. This parameter plays an important role in the structure and division of communities, as higher values tend to result in a greater number of communities, but smaller in size, while lower values can direct us to a smaller number of communities, but larger in size. The default value proved to be more appropriate as it approximated the number of jurisdictions.

Modularity-based methods are computationally more efficient and easier to parallelize compared to other approaches such as Bayesian ones [36]. This makes modularity a more feasible choice for analyzing large-scale networks, such as the ones studied in this work.

### Computing the fractal dimension of networks

We use the Maximum-Excluded-Mass-Burning (MEMB) algorithm to perform a box covering to calculate the fractal dimension of the MSTs of each community. In a box-counting method, the goal is to determine how the number of distinct “boxes” needed to cover the structure scales with the size of the boxes.

For very large networks, such as the MSTs of the large communities, we use the Compact-Box-Burning (CBB) algorithm, which is more efficient. Employing a breadth-first search, it generates a box by growing it from one randomly selected node towards its neighborhood until the box is compact, or equivalently that each box should include the maximum possible number of nodes. In our experiments, the initial size of a box within the CBB algorithm begins at 2. Both algorithms are explained in [37] and [38]. Both are available at (https://hmakse.ccny.cuny.edu/software-and-data/).

## Results

**Fig 2:**
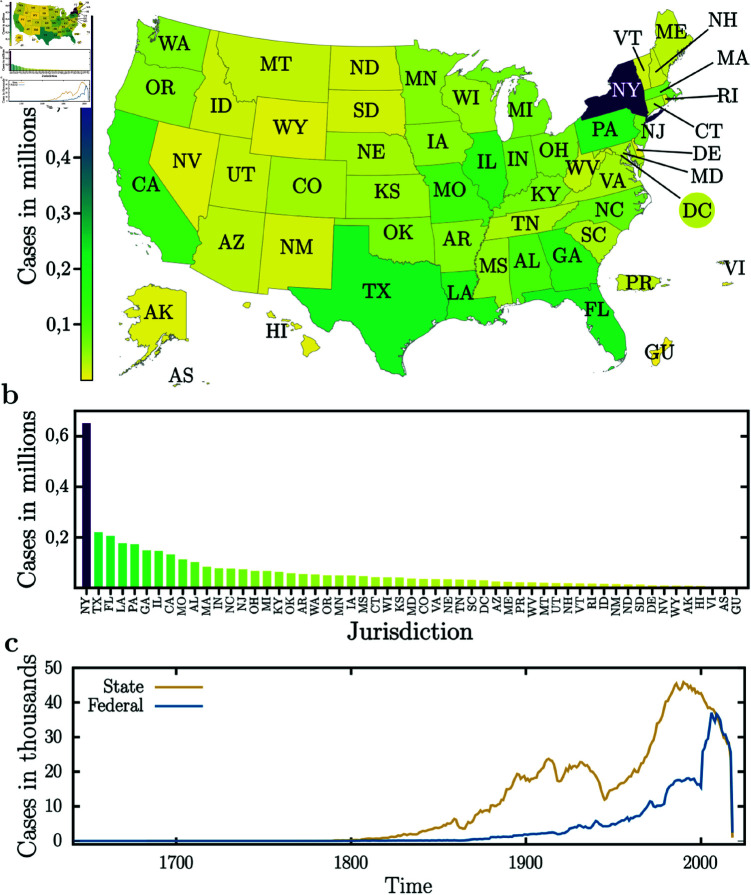
CAP data characterization. **(a)** Here we show the heat-map of US court cases grouped by State jurisdiction. The color represents the number of cases within each jurisdiction according to the color scale shown on the left of the figure. **(b)** Sorted bar chart of the number of cases for each jurisdiction. **(c)** Evolution of number of cases over the years. This figure shows that the number of cases in the 20th century grew by almost 6 times compared to the 19th century cases.

First, we performed an exploratory analysis of the CAP dataset. In [sec:methods:cap]Caselaw access project, we show the huge volume of data we are using. Additionally, we performed an analysis to understand how data is distributed geographically, across federal and non-federal jurisdictions, over time, and across courts. In Figure 2, we show the results of these analyses. In Fig 2a, we show how the cases are distributed across the American states. As depicted in Fig 2b, cases are distributed unevenly across states, with only a few states having more than 500,000 cases. In Fig 2c, we observe that the number of cases in the 20th century grew almost 6 times compared to the 19th century. Furthermore, we also note that there are approximately 3.7 million state jurisdiction cases and approximately 1.3 million federal jurisdiction cases, which makes state jurisdiction cases represent more than 2/3 of the total number of legal cases.

Based on this data, we built networks of legal cases, as described in [sec::the_models]Network models, of which we will show some results next. Precisely, we analyzed the *citation network* and the *bibliographic coupling network* to understand how citation relationships between the legal cases are internally organized. The obtained citation network consists of a directed network with 5,084,607 vertices and 45,532,896 edges from which we calculate traditional complex network metrics. We also calculated metrics for the bibliographic coupling network composed of 5,084,607 vertices and 11,487,693,779 undirected edges. The results obtained for these two networks are shown side by side in Table 2. As shown in this table, the citation network has 5,039,941 vertices in the *Largest Weakly Connected Component* (LWCC), which corresponds to approximately 99% of the network’s vertices. We also observe that the network is very sparse since the average density is $\approx 1.79\;\cdot \;10^{-6}$. Regarding to *bibliographic coupling network*, we observe a greater number of edges with an average edge density of $\approx 1.06\;\cdot \;10^{-3}$, which is 592 times higher than that for the citation network. According to the literature [30, 39], the edges modeled by the bibliographic coupling network may provide a more accurate index in capturing relationships between documents dealing with the same subject than the edges modeled by the citation network, since they produce identifications among the citing documents and not only among the cited ones. The combination of both can lead to future visualizations of both relationships through specific applications. Therefore, we will further characterize this network in the following and use it in our analyses henceforth.

**Table 2:** Basic citation and bibliographic coupling network metrics.

Metrics	Citation	Bibliographic Coupling
Vertices	5,084,607	5,039,941
Edges	45,532,896	11,487,693,779
Vertices in LWCC	5,039,941	-
Edges in LWCC	45,440,742	-
Vertices in LCC	-	4,655,666
Edges in LCC	-	11,487,629,415
Density	$1.79\cdot 10^{-6}$	$1.06\cdot 10^{-3}$
Average clustering coefficient	0.0806	-
Directed	yes	no
Weighted	no	yes

**Fig 3:**
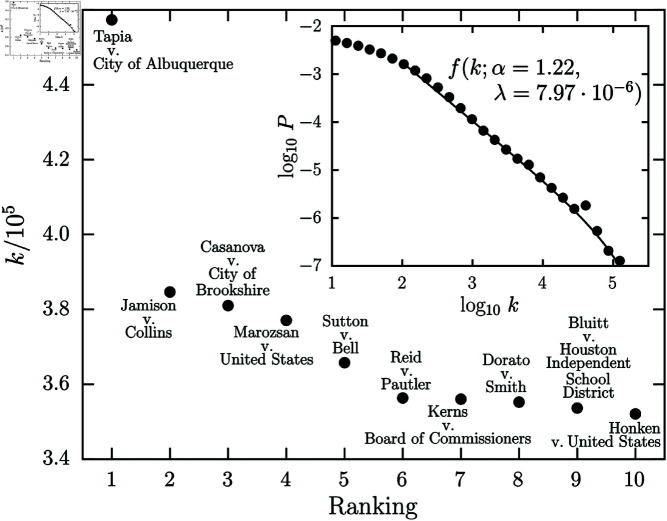
Degree of vertices in the bibliographic coupling network. Here, we show the ranking plot of the vertices in the bibliographic coupling network. Furthermore, we also highlight the vertices with the highest degree in this network. In the inset, we show the degree distribution of the vertices (black circles) following a power law with exponential cutoff (solid line) $f(k;\alpha ,\lambda )\propto x^{-\alpha }\exp(-\lambda x)$, where α=1.22 and $\lambda =7.97\cdot 10^{-6}$ are the best-fit values obtained through MLE.

The statistical analysis of the degree of the *bibliographic coupling network* vertices sheds light on the role of the “hubs” of this network. Fig 3 shows the ranking plot and the degree distribution of the bibliographic coupling network, and the vertices highlighted in Fig 3 are those that are most connected. As shown in Fig 1, for an edge to exist between two vertices in the bibliographic coupling network, they must cite a same set of legal cases. In this sense, the vertices shown in Fig 3 are those that cite frequently-cited cases and are therefore presented as standard models for citing legal cases (e.g., landmarks). Therefore, these hubs and their edges help to maintain the cohesion between similar vertices, forming an internal structure that connects all parts of the network, much like the circulatory system of living organisms [9]. In the inset of Fig 3, we show the degree distribution of the vertices from the bibliographic coupling network obtained through a logarithmically binned histogram. We used a python package designed for analysis of heavy-tailed distributions [40] in order to find the best candidate among the following distributions frequently found in empirical data: power law, lognormal, exponential, power law with exponential cutoff, and stretched exponential (Weibull). We performed the *Loglikelihood Ratio Test* and a power law with exponential cutoff $f(k;\alpha ,\lambda )\propto k^{-\alpha }\exp(-\lambda k)$ was the best description available for our data, with the parameters $x_{\text{min}}=122$, α=1.22 and $\lambda =7.97\;\cdot \;10^{-6}$ obtained through Maximum Likelihood Estimation (MLE).

The main difference in the figures refers to the number of edges. Any processing in the Bibliographic Network is not trivial due to its size.

**Fig 4:**
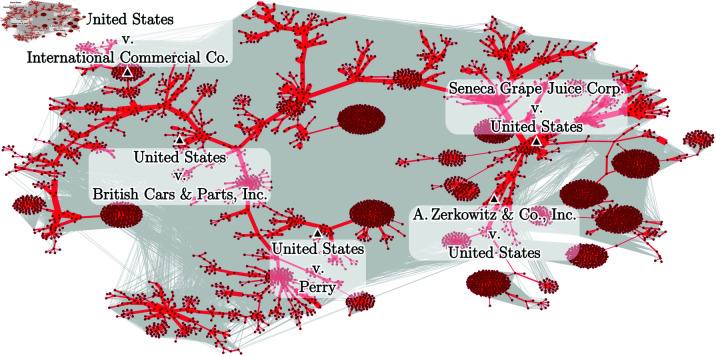
Illustration of a typical community found in the bibliographic coupling network. The dark red dots represent the vertices (legal cases) of the community, and the light gray lines represent the edges between those vertices in the bibliographic coupling network. The black triangles represent the vertices with the highest degree (hubs), and the red lines are the MST edges of that community. The thicker the MST edges, the greater the weight of that edge in the bibliographic coupling network. That is, the greater the similarity between the cases cited by the vertices (legal cases) connected to that edge.

*Community analysis* deepened our understanding of the bibliographic coupling network’s internal structure. Utilizing the *Louvain algorithm* [33], we detected 83 communities. Next, we compute the *Maximum Spanning Tree* (MST) for each community. In Fig 4, we show an illustration of a typical community found in the bibliographic coupling network. In this figure, we can see how dense these structures are, which makes a direct analysis of fractal dimensions challenging. To address this, we calculated the Maximum Spanning Tree (MST) of each community. An MST provides a simplified representation of the network’s structure, acting as its “backbone” by preserving all nodes while reducing the network to a non-fragmented tree structure. This ensures that the essential connectivity of the community is maintained while enabling meaningful fractal analysis.

We then estimate the *fractal dimension* of the communities using the *Compact-Box-Burning* (CBB) algorithm [38]. Formally, the *fractal dimension* is derived from the *Hausdorff–Besicovitch dimension* of a fractal object, which is mathematically related to the way in which the *measure* of a set depends on the *metric* of the space in which it is embedded [41]. In the context of complex networks, it is strongly related to how the number of vertices grows with distance in the network [37].

**Fig 5:**
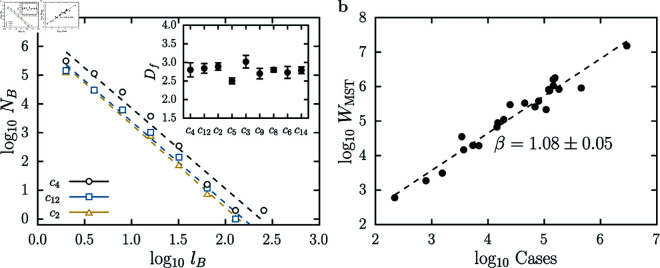
Self-similarity analysis for the MSTs of coupling network communities. **(a)** Log-log plot of the number of boxes (*N*_*B*_) as a function of the size of the boxes (*l*_*B*_) obtained from the CBB algorithm. In this figure, we show a power-law dependence $N_{B}\propto l_{B}^{-D_{f}}$ for three of the largest communities. We were unable to obtain a good estimate of *D*_*f*_ for the largest community (*c*_0_) due to computational limitations (this community has approximately 2.9 million vertices!). For the second largest community (*c*_4_), we find $D_{f}=2.80\;\pm \;0.19$ as shown in the figure. The remaining communities showed similar behavior with $D_{f}(c_{12})=2.84\;\pm \;0.13$, $D_{f}(c_{2})=2.89\;\pm \;0.10$, $D_{f}(c_{5})=2.50\;\pm \;0.08$, $D_{f}(c_{3})=3.02\;\pm \;0.17$, $D_{f}(c_{9})=2.70\;\pm \;0.14$, $D_{f}(c_{8})=2.80\;\pm \;0.06$, $D_{f}(c_{6})=2.73\;\pm \;0.16$ and $D_{f}(c_{14})=2.79\;\pm \;0.09$. In the inset, we show the estimates for the fractal dimensions for the largest communities. This result is a strong evidence that this structure presents a self-similar geometry. **(b)** Log-log plot of the total weight of the MST (*W*_*MST*_) as a function of the number of legal cases in the community. Here, we also show a power-law dependence of the total weight of MSTs on the number of community cases. These two quantities scale with a super-linear exponent β=1.08±0.05.

In Fig 5, we show a self-similarity analysis for the MSTs generated from the coupling network communities. In Fig 5a, we show the plot of the number of boxes *N*_*B*_ as a function of the size of the boxes *l*_*B*_ which is used to obtain the fractal dimension *D*_*f*_ of the MST of some communities. In order to obtain *N*_*B*_, we used the CBB algorithm cited previously and found values of *D*_*f*_ that are numerically close, all ranging between 2.50 and 3.02. In the inset of this figure, we show the estimates for the fractal dimensions for the largest communities. Here, due to computational limitations, we are computing the fractal dimension using just one realization of the CBB algorithm. We emphasize that this approach does not change our results, since the CBB algorithm is not sensitive to the specific realization used [38]. In Fig 5b, we show the scaling of the MST’s total weight as a function of the number of legal cases in the community. As a result, we found that these two quantities scale with a weakly, but super-linear exponent β=1.08±0.05. These two results indicate that there is a intricate fractal structure within the community. The consistent self-similar nature of the MST’s might have implications for the dynamics of the US Caselaw. For instance, it could influence how legal doctrines or decisions spread and evolve within the network. In living organisms, the fractal structure of a vascular system impacts energy distribution and transport, which can influence allometric relationships concerning metabolic rate and organism size [9]. We will later discuss a similar phenomenon observed in the US Caselaw.

**Fig 6:**
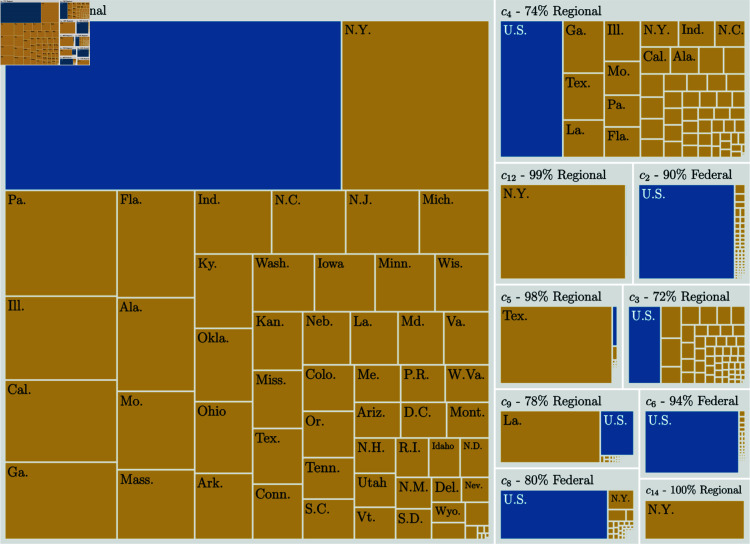
Treemap displaying jurisdiction composition in the top-10 communities. This figure shows the composition of the ten largest communities from the perspective of jurisdiction. Blue color corresponds to Federal jurisdiction and orange to State jurisdiction. Note that, except for communities *c*_0_, *c*_3_, *c*_4_ and *c*_9_, there is a predominance of single jurisdiction in the communities and most of them are regional.

In Fig 6, we present a treemap illustrating the jurisdictional composition of the 10 largest communities out of the 83 identified in the bibliographic coupling network. A detailed list of the abbreviations used in Fig 6, along with the total number of cases for each jurisdiction, is provided in the S1 Tab. All the communities are formed by grouping cases based on their similarity, which is determined through citation co-occurrences. Considering that the United States is a federation where states have significant autonomy, it is reasonable to hypothesize that these communities may, to some extent, reflect the geographical distribution of cases across jurisdictions. By examining the communities from a jurisdictional perspective—whether federal or state—we aim to explore the influence of regional and federal nature of the cases on community formation and better understand their role in shaping the network’s structure.

Interestingly, our analysis reveals that jurisdiction alone is not the sole factor driving community formation. Of the 83 communities detected, only five are composed exclusively of cases from a single jurisdiction: two federal communities with fewer than 45,000 cases each, and three state communities with fewer than 80,000 cases each. The majority of communities exhibit a mixed composition, indicating that other factors, such as legal themes or citation patterns, also contribute to their structure.

However, jurisdiction does play a significant role in shaping the communities. This is evident from the fact that the largest communities tend to show a predominance of cases from either federal or state jurisdictions. For example, communities *c*_2_, *c*_8_, and *c*_6_ are primarily federal, whereas *c*_0_, *c*_4_, *c*_12_, *c*_5_, *c*_3_, *c*_9_, and *c*_14_ are dominated by state cases (see Fig 6). This balance between mixed and jurisdictionally dominant communities highlights the interplay of regional, federal, and thematic factors in the formation of the bibliographic coupling network.

**Fig 7:**
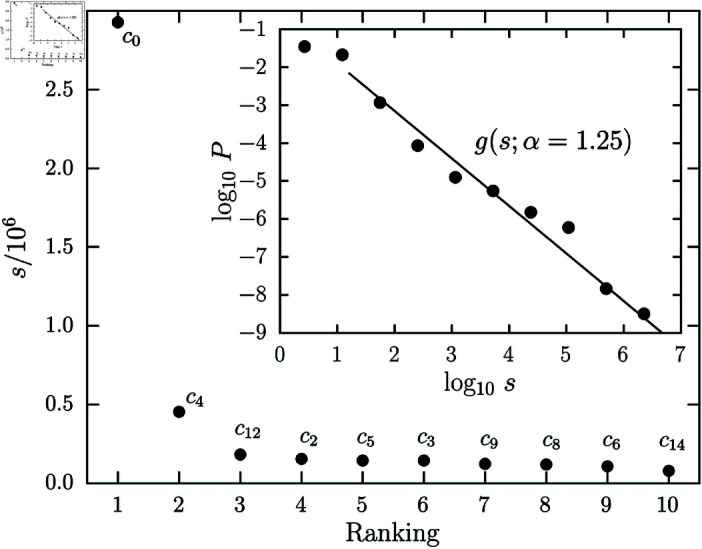
Communities size for bibliographic coupling network. Here, we show the communities size ranking plot for the bibliographic coupling network. In the inset, we show the community size distribution (black circles) characterized by an asymmetric shape following power-law distribution (solid line) $g(s;\alpha )\propto s^{-\alpha }$, where α=1.25 is the best-fit value obtained through MLE. As observed, the size of the communities decreases rapidly, with the largest community in the network being ≈6 times larger than the second.

Fig 7 shows the ranking plot and size distribution of communities for the *bibliographic coupling network*. The size of communities decreases very fast. Basically, there are few large communities and a large number of small communities. In the inset of Fig 7, we show the community size distribution for the bibliographic coupling network obtained through a logarithmically binned histogram. Here, similarly to what was done to the degree distribution of the vertices, we performed the *Loglikelihood Ratio Test* and a power law $g(s;\alpha )\propto s^{-\alpha }$ was the best description available for our data, with $x_{\text{min}}=14$ and *alpha* = 1.25 obtained through Maximum Likelihood Estimation (MLE). The patterns in Figs 3 and 7 align with Pareto’s principle, showing that a few vertices dominate edge connections, and a minority of communities contain most legal cases. This recurs even in smaller system fractions, resembling the inherent link between scale invariance and self-similarity seen in biological ecosystems [42].

**Fig 8:**
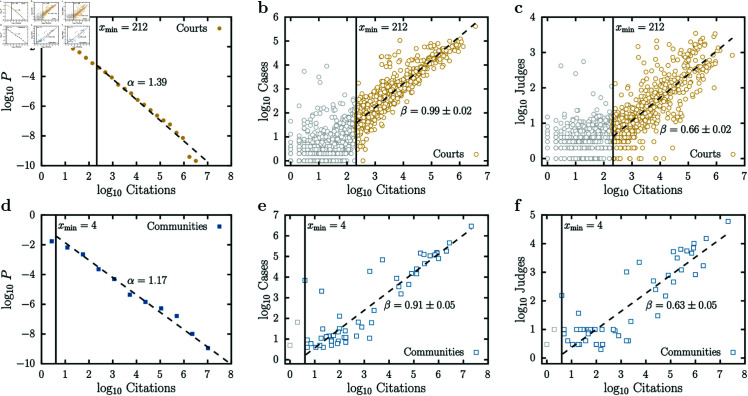
Citation distribution and allometric relations for the bibliographic coupling network. **(a)** Citation distribution, for courts. **(b)** Cases (size) as function of citations (mass), for courts. **(c)** Number of judges as function of citations, for courts. **(d)** Citation distribution, for communities. **(e)** Cases (size) as function of citations (mass), for communities. **(f)** Number of judges as function of citations, for communities. In Figures (a) and (d), we show that citations follow a power-law distributions for both courts and communities. In order to estimate exponent α and the cutoff $x_{\text{min}}$ of the power laws, we applied the MLE algorithm described in [43] and found α=1.39 and $x_{\text{min}}=212$ for courts, and α=1.17 and $x_{\text{min}}=4$ for communities. In Figure (b), we show that, for courts, cases depend on citations with a linear relation (β=0.99±0.02), while in (e) they depend with “almost”-linear relation (β=0.91±0.05). Figures (c) and (f) can be interpreted as the allometric law between metabolic rate and mass for the US Caselaw, where the number of judges would be related to the metabolic rate and the number of citations to the mass of the system. We found that a sub-linear exponent applies to both courts (β=0.66±0.02) and communities (β=0.63±0.05), regardless of the way the “organism” is defined, evincing a characteristic behavior of living organisms.

Viewing the US Caselaw as an evolving entity via the bibliographic coupling network (Fig 2) and acknowledging its scale invariance (Figs 3 and 7), we conducted an allometric analysis to explore potential operational economies of scale. In Fig 8a and 8d, we show that citations follow power-law distributions for both courts and communities. We estimated the exponent α and the cutoff $x_{\text{min}}$ for both power laws shown in these figures using the *Maximum Likelihood Estimation* (MLE) algorithm described in [43]. By doing this, we found α=1.39 and $x_{\text{min}}=212$ for courts, and α=1.17 and $x_{\text{min}}=4$ for communities. This algorithm outperforms the ordinary least squares method in providing more accurate estimates for the parameters of power-law distributions. Moreover, it allows us to define the cutoff point from which we should estimate allometries, as referenced in [44]. Additionally, the allometric relations for the bibliographic coupling network are shown in Fig 8. Each data point represents a court in Fig 8b and 8c, while in Fig 8e and 8f represents a community. Furthermore, in Fig 8b and 8e, we show that cases depend on citations with a nearly linear relation. Remarkably, as shown in Fig 8c and 8f, we discovered an allometric relationship between the number of judges and citations, featuring a sublinear exponent of approximately 2/3. This suggests a close analogy between the organizational structures of the US Caselaw and those of living organisms [9], where the numbers of citations and judges would correspond to the masses and the metabolic rates, respectively. Under the same framework, the sublinear behavior indicates a potential *economy of scale* in the growth of the US Caselaw.

The asymmetries observed in the scaling relationships—such as the degree distribution, the rank-size relationship of communities, and the allometric scaling between judicial activity and case volume—indicate that the bibliographic coupling network operates under emergent, non-random principles that organize its structure and dynamics. Ultimately, the importance of these scaling relationships lies in what they reveal about the nature of the US Caselaw itself: a self-organizing, adaptive, and interconnected structure, much like a living ecosystem.

## Conclusion

We have modeled the US legal system through citation and bibliographic networks, encompassing over 5 million cases across 360 years. Our study’s innovation lies in uncovering a sublinear allometric relation between citation counts and judges in legal case communities. This discovery aligns with models describing how living organisms transport essential materials through space-filling fractal-like structures for survival. The fractal nature of community Maximum Spanning Trees (MSTs) offers a tentative explanation for this relationship, suggesting a structural and functional correspondence between the US Caselaw network and biological systems.

Legal cases within each community generally share or relate to the same topics. While society’s development drives case numbers, judges generate citations. In this biological metaphor, a community is the animal, citations represent mass, and the number of judges symbolizes metabolism. Just as fractal networks efficiently distribute resources throughout an organism, our findings imply that the US legal system may have evolved a similarly efficient structure for disseminating legal information and precedent. This correlation not only provides insight into legal network dynamics but also adds a novel dimension to our understanding of legal organic structures as complex systems.

Moreover, the resemblance to space-filling fractal networks in living organisms emphasizes the adaptability and resilience of the legal system, capable of expanding and contracting in response to societal needs and pressures. Our results therefore suggest the legal network has developed mechanisms for efficient information transfer and resource allocation, ensuring its survival and functionality over centuries. This study opens new avenues for interdisciplinary research, inviting further exploration into the universal principles governing complex systems, whether in the realm of biology, cities or jurisprudence.

Although our study uses quantitative methods to highlight legal patterns and trends, we do not adopt a positivist stance that equates the methodologies and categories of the social sciences with those of the life sciences or mathematics. We recognize the potential of life pattern studies in elucidating shifts in legal decisions over time, but we also claim the importance of integrating these findings with qualitative insights from Legal History. This article aims to contribute to, rather than definitively conclude, the discourse on court behavior and judicial values across history.

We emphasize that our work is not the first to draw parallels between biological and social systems. As already mentioned, in 2007, Bettencourt *et al*. [10] extended the ideas behind biological allometries to urban environments, finding that US cities exhibit sub-linear allometric relations with population sizes, implying economies of scale in urban indicators as the per capita measurement decreases with population size. This precedent reinforces the validity and potential of cross-disciplinary analogies to uncover underlying principles in complex systems.

Future research on the parallels between legal networks and biological systems should focus, at least, on four main areas. First, investigate the evolutionary dynamics of legal networks by examining the adaptation and “genetic drift” of legal doctrines over time, mirroring biological evolution. Second, analyze the complexity and resilience of the legal network by assessing how it responds to shocks, such as landmark cases, to understand its “regulatory mechanisms” and stability akin to biological responses to environmental stressors. Third, conduct comparative studies between the legal networks of different countries, particularly Brazil’s (based on Civil Law), to discern how cultural and legal traditions influence network structures and to identify universal patterns or unique adaptations in the legal system. Finally, we also aim to explore the citation network to understand the growth processes that occur within the network structure and investigate in detail the evolutionary dynamics of communities.

## Supporting information

S1 Table List of abbreviations and total cases by jurisdiction (PDF)

S1 Fig Outline of case structure in Caselaw. (PDF)

S2 Fig Frequency of name variations for collegiate decisions in the Caselaw Access Project. (PDF)

S1 File Regular expressions (Regex) for judges name extraction. (PDF)
