# Supplementary material for: The US Caselaw as a living system
Source: PLoS One. 2025 May 23;20(5):e0324386. doi: 10.1371/journal.pone.0324386 (PMC12101733; doi:10.1371/journal.pone.0324386)
Supplement: S1 Table — (PDF) [file pone.0324386.s001.pdf]

S1 Tab. List of abbreviations and total cases by jurisdiction.

Jurisdiction	Abbreviation	Total Cases
Alabama	Ala.	98,933
Alaska	Alaska	10,094
American Samoa	Am. Samoa	854
Arizona	Ariz.	26,158
Arkansas	Ark.	53,219
California	Cal.	53,219
Colorado	Colo.	34,570
Connecticut	Conn.	42,274
Dakota Territory	Dakota Territory	271
District of Columbia	D.C.	28,680
Delaware	Del.	11,356
Florida	Fla.	196,950
Georgia	Ga.	135,874
Guam	Guam	137
Hawaii	Haw.	8,443
Idaho	Idaho	18,408
Illinois	Ill.	135,543
Indiana	Ind.	73,165
Iowa	Iowa	44,707
Kansas	Kan.	39,744
Kentucky	Ky.	54,702
Louisiana	La.	155,973
Massachusetts	Mass.	77,940
Maryland	Md.	35,713
Maine	Me.	23,387
Michigan	Mich.	62,048

(continue on next page)

– (continued from previous page)

Jurisdiction	Abbreviation	Total Cases
Minnesota	Minn.	47,157
Mississippi	Miss.	44,853
Missouri	Mo.	109,107
Montana	Mont.	21,381
Navajo Nation	Navajo Nation	358
North Carolina	N.C.	69,203
North Dakota	N.D.	15,466
Nebraska	Neb.	33,004
Nevada	Nev.	10,647
New Hampshire	N.H.	19,113
New Jersey	N.J.	61,621
New Mexico	N.M.	17,077
Northern Mariana Islands	N. Mar. I.	294
New York	N.Y.	590,757
Ohio	Ohio	59,109
Oklahoma	Okla.	55,975
Oregon	Or.	48,876
Pennsylvania	Pa.	161,139
Puerto Rico	P.R.	21,175
Rhode Island	R.I.	17,793
South Carolina	S.C.	28,478
South Dakota	S.D.	14,828
Tennessee	Tenn.	29,912
Texas	Tex.	204,957
Tribal Jurisdictions	Tribal	558
United Kingdom	U.K.	1

(continue on next page)

– (continued from previous page)

Jurisdiction	Abbreviation	Total Cases
United States	U.S.	1,279,451
Utah	Utah	20,076
Virginia	Va.	33,331
Virgin Islands	V.I.	3,147
Vermont	Vt.	17,241
Washington	Wash.	50,938
Wisconsin	Wis.	41,241
West Virginia	W. Va.	22,593
Wyoming	Wyo.	10,246

The abbreviations shown here are standard abbreviations found in the *Caselaw Access Project* and correspond to the same abbreviations used in the Fig. 5 of the main article.
