# Supplementary material for: The US Caselaw as a living system
Source: PLoS One. 2025 May 23;20(5):e0324386. doi: 10.1371/journal.pone.0324386 (PMC12101733; doi:10.1371/journal.pone.0324386)
Supplement: S1 Fig — (PDF) [file pone.0324386.s002.pdf]

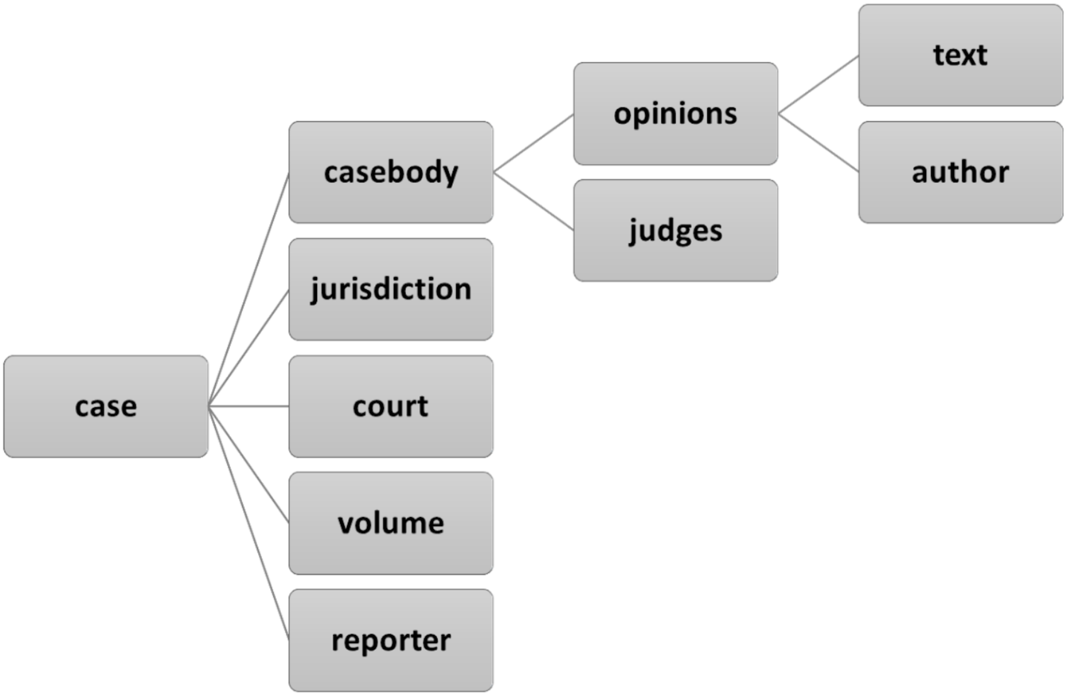

S1 Fig. Outline of case structure in Caselaw. In this figure, we see the structure of a typical document from Caselaw Access Project. Here, we observe that the name of the judges in the cases are typically in the Subsections Judges and Opinion.
