# Supplementary material for: The US Caselaw as a living system
Source: PLoS One. 2025 May 23;20(5):e0324386. doi: 10.1371/journal.pone.0324386 (PMC12101733; doi:10.1371/journal.pone.0324386)
Supplement: S2 Fig — (PDF) [file pone.0324386.s003.pdf]

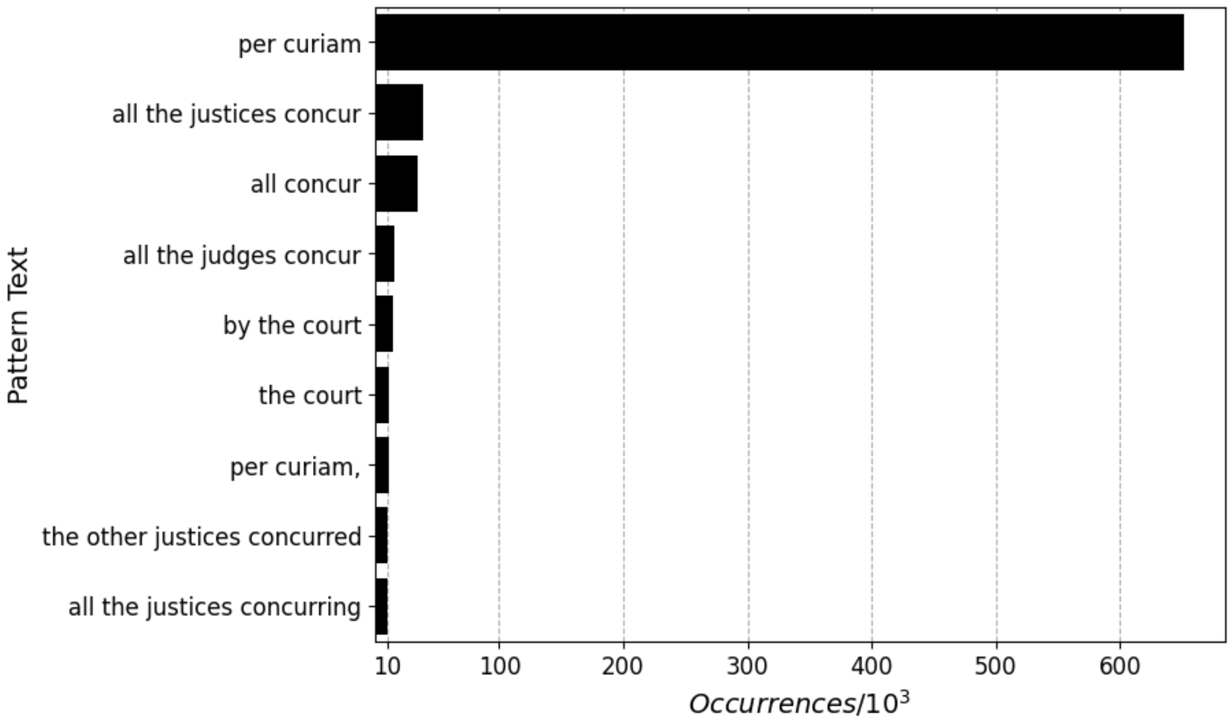

S2 Fig. Frequency of name variations for collegiate decisions in the Caselaw Access Project. Here we can see that the term "per curiam" is by far the most frequent for collegiate decisions.
