# Supplementary material for: The US Caselaw as a living system
Source: PLoS One. 2025 May 23;20(5):e0324386. doi: 10.1371/journal.pone.0324386 (PMC12101733; doi:10.1371/journal.pone.0324386)
Supplement: S1 File — (PDF) [file pone.0324386.s004.pdf]

## S1 File. Regular Expressions (Regex) for Judges Name Extraction

- *Names before common judge functions separated by 'and':* Captures names of judges that precede titles or common functions, considering variations in the spelling of the functions and can be separated by "and".

```
^([^,]+) and ([^,]+),\s*(?:circuit|district|judge|justice|
   ↪ commissioner|circuit judge|judges|president|chief
   ↪ judge|district judge|senior judge|circuit judge|
   ↪ commissoner|county judge|retired justice|)\b
```

- *Names before common judge functions:* Captures names of judges that precede titles or common functions, considering variations in the spelling of the functions.

```
^([^,]+),\s*(?:circuit|district|judge|j|justice|
   ↪ commissioner|circuit judge|judges|president|chief
   ↪ judge|district judge|senior judge|circuit judge|
   ↪ commissoner|county judge|retired justice|)
```

- *Names before specific acting indications:* Targets names that precede acting indications such as "acting", "presiding", or "special" judges.

```
^([^,]+),\s*(?:before|opinion|acting|presiding|special)
```

- *Names with title abbreviations:* Focused on names that precede common abbreviations of judge titles, such as "j", "cj", or "pj".

```
^([^,]+),\s*(?:j|cj|pj|aj|sj|ch j|c j|jj|acj|vcj|j c c|dj|
   ↪ srj|ej|)\b
```

- *Names of multiple judges separated by 'and' with title abbreviation:* Identifies and separately extracts the names of multiple judges listed in the same sentence, separated by the conjunction "and".

```
^([^,]+) and ([^,]+),\s*(?:j|cj|pj|aj|sj|ch j|c j|jj|acj|
   ↪ vcj|j c c|dj|srj|ej|)\b
```

- *Judges with formal titles:* Captures judges who follow formal titles such as “chief justice”, “mr justice”, “judge”, or “commissioner”.

```
^(chairman|chancellor|bv judge|by judge|by justice|by mr
  ↪ justice|by|air justice|before judge|a j|a b|a c|b f|
  ↪ cb j|ch j|mr chiefjustice|me justice|el juez asociado
  ↪ |mr chief justice|juez|chiee justice|chief justice|mk
  ↪  justice|mr chiee justice|ml justice|ms justice|mn
  ↪ justice|mr justice|judge|commissioner|senior judge|
  ↪ president judge|absent|absent judge|absente|acting
  ↪ justice|mr chief justioe|mr chief justrice|mr
  ↪ chiefjustice|mr chief jutstice|mr chiep justice|mr
  ↪ chiej justice|mr chief|justice)\s+([a-z]+(?:\s[a-z]+)
  ↪ *)
```

- *Judge names with common functions without comma separation:* Capture names that are immediately followed by judge titles, with no other words or characters between them, except for spaces

```
^\b([A-Za-z]+(?:\s[A-Za-z]+)*)\s(?:j|cj|pj|aj|sj|ch j|c j|
  ↪ jj|acj|vcj|j c c|dj|srj|ej|circuit|district|judge|
  ↪ justice|commissioner|circuit judge|judges|president|
  ↪ chief judge|district judge|senior judge|circuit judge
  ↪ |commissoner|county judge|retired justice)\b
```
